# Resistance gene mutations and phylogenetic relationships in *Candidozyma auris* isolates from Russia

**DOI:** 10.3389/fcimb.2026.1929119

**Published:** 2026-08-28

**Authors:** Ellina Grigorievna Oganesyan, Anna S Zhuk, K. V. Gusarova, S. V. Kovyrshin, E. S. Alekseevskaya, O. E. Orlova, S. N. Khostelidi, O. P. Kozlova, A. V. Liubimova, A. E. Taraskina, Natalia Vsevolodovna Vasilyeva

**Affiliations:** 1 North-Western State Medical University named after I.I. Mechnikov, Ministry of Health of the Russian Federation, St. Petersburg, Russia; 2 Institute of Applied Computer Science, ITMO University, St. Petersburg, Russia; 3 Institute of Cytology, Russian Academy of Sciences, St. Petersburg, Russia; 4 Moscow Scientific and Practical Center for Laboratory Research, Moscow, Russia

**Keywords:** antifungal resistance, *Candidozyma auris*, clade I, FCY2, genomic epidemiology

## Abstract

**Introduction:**

*Candidozyma auris* is an emerging healthcare-associated fungal pathogen with a high propensity for nosocomial transmission and development of antifungal resistance. This study aimed to identify resistance-associated genomic variants and characterize the phylogenetic structure of clinical *C. auris* isolates circulating in Russia.

**Methods:**

We analyzed 82 isolates collected between 2017 and 2023 from 18 hospitals in the Northwestern and Central Federal Districts of the Russian Federation. Antifungal susceptibility testing was combined with whole-genome sequencing, targeted *FCY2* sequencing, and comparative phylogenomic analysis using publicly available international genomes.

**Results:**

All isolates analyzed in this study belonged to clade I and showed a highly conserved profile of elevated azole MICs. The consistent detection of *ERG11* (K143R), *TAC1B* (A640V), and *CDR1* (V704L) suggests that reduced azole susceptibility in this population is associated with both target-gene alteration and efflux-mediated mechanisms. All isolates remained susceptible to echinocandins in vitro, and no resistance-conferring mutations were detected in *FKS1*, consistent with the absence of an echinocandin-resistant phenotype. Decreased susceptibility to flucytosine was mainly associated with the *FCY2* (L383*) nonsense mutation, which was confirmed by targeted Sanger sequencing in additional isolates. Phylogenomic reconstruction showed that the Russian isolates represented a restricted segment of global clade I diversity and revealed two major geographically structured lineages corresponding to two large metropolitan areas in European Russia.

**Discussion:**

The distribution of closely related isolates across hospitals supports local persistence and inter-hospital dissemination of genetically related strains. These findings provide important insights into the molecular epidemiology, antifungal resistance mechanisms, and transmission dynamics of *C. auris* in Russia.

## 1 Introduction

*Candidozyma auris* (previously *Candida auris*) has emerged over the past decades as a major global health threat due to its capacity to cause invasive healthcare-associated infections and outbreaks, often involving high rates of antifungal resistance and treatment failure (Satoh et al., 2009; Lockhart et al., 2017; Liu et al., 2024). Since its first description in 2009, *C. auris* has been reported on all continents except Antarctica, demonstrating an ability to spread between patients and persist in hospital environments (Rhodes and Fisher, 2019). The growing prevalence of multidrug-resistant *C. auris* complicates clinical management and infection control, highlighting the urgent need for local and global surveillance (Jones et al., 2024; CDC, 2025).

One of the most concerning aspects of *C. auris* is its frequent resistance to commonly used antifungal agents, including azoles and, less frequently, echinocandins, polyenes, and flucytosine (Achilonu et al., 2025). Although point mutations in target genes are known to contribute to antifungal resistance, regional differences in mutation patterns remain poorly understood (Sanyaolu et al., 2022; Suphavilai et al., 2024). To date, six distinct clades of *C. auris* have been identified, each with a defined geographic distribution and markedly divergent resistance profiles, making the study of regional strain diversity particularly relevant (Suphavilai et al., 2024; Davi Marena et al., 2025). Genomic evidence indicates that *C. auris* isolates from distinct phylogenetic clades may harbor sets of mutations conferring resistance to antifungal agents. For instance, clade I (South Asian) isolates frequently carry *ERG11* substitutions Y132F and/or K143R, which directly contribute to elevated fluconazole minimum inhibitory concentrations (MICs) and are commonly reported in outbreak settings (Jangir et al., 2023). Clade IV (South American) strains have independently acquired the same Y132F substitution, while clade III (African) isolates typically harbor an alternative *ERG11* (F126L) mutation instead of Y132F/K143R, reflecting a distinct evolutionary pathway (Williamson et al., 2022). In contrast, clades II (East Asian), V (Iranian), and VI (Indomalayan) usually retain wild-type *ERG11* sequences and correspondingly show lower fluconazole MICs (Khan et al., 2024). These inter-clade differences suggest that resistance in *C. auris* has evolved under distinct regional selective pressures, with each clade accumulating mutations largely independent of the others.

Beyond *ERG11*, mutations in other resistance genes also show clade-specific distributions. The transcription factor gene *TAC1B*, which regulates the efflux pump *CDR1*, has been implicated in azole resistance: the *TAC1B* A640V variant is prevalent in highly fluconazole-resistant clade I isolates and often co-occurs with *ERG11* K143R. In clade IV, a distinct in-frame deletion (*TAC1B* F862_N866del) has been reported as a marker of resistance in certain isolates (Rybak et al., 2020; Barker et al., 2025). Although most clade I isolates carry either the Y132F or K143R substitution in *ERG11*, these variants are not uniformly distributed within the clade. Both substitutions were originally identified in South Asian isolates from India and Pakistan, often co-occurring in the same collections (Lockhart et al., 2017; Rhodes and Fisher, 2019). In a subsequent global genomic survey of 304 isolates, Y132F was the more widespread variant, detected across 11 countries in approximately 53% of clade I isolates, whereas K143R was concentrated within a specific clade I sublineage and accounted for around 43% of isolates (Chow et al., 2020). Additionally, the *TAC1B* A640V mutation, a well-known efflux-regulator polymorphism, is common in clade I: for example, New York outbreak lineages showed near-universal A640V (Burrack et al., 2022), yet other *TAC1B* alterations are also encountered; notably, the R495G substitution has been identified in a single clade I isolate, and F214S has been reported in two clade II isolates and one clade IV isolate (Rybak et al., 2020). Thus, further characterization of resistance mutations, taking into account their clade-specific and geographic variability, is essential for understanding local epidemiological patterns and informing rational approaches to antifungal therapy of *C. auris*.

Despite the increasing amount of international genomic data, the molecular epidemiology of *C. auris* in Russia remains poorly characterized. The first whole-genome sequencing data for Russian *C. auris* were obtained from a strain recovered from candidemia in 2017. Genomic analysis showed that this isolate belonged to clade I and likely represented an independent introduction from South Asia (Pchelin et al., 2020). Our previous study based on short tandem repeat (STR) genotyping further revealed that all known Russian isolates belong to clade I and suggested local transmission within healthcare settings (Oganesyan et al., 2025). Nevertheless, the full spectrum of mutations in antifungal resistance genes and the phylogenetic position of Russian isolates within the global *C. auris* population have not yet been examined using whole-genome sequencing.

In the present study, we performed whole-genome sequencing and targeted analysis of the *FCY2* gene in a collection of Russian *C. auris* isolates to identify resistance-associated mutations and characterize population structure. By integrating local and international datasets, we aimed to place Russian isolates in a broader phylogenetic framework and assess their genetic diversity relative to other clade I strains worldwide. In addition to identifying resistance mechanisms and population structure, we also examined the evolutionary background of the *C. auris* population represented by our Russian isolate collection by estimating the time to the most recent common ancestor (TMRCA) of the sampled isolates. This analysis provides a temporal perspective on the persistence of related genotypes in healthcare settings, which may inform genomic surveillance and infection control strategies.

## 2 Materials and methods

### 2.1 Isolates and culture conditions

A total of 82 clinical *C. auris* isolates were collected between 2017 and 2023 from 18 hospitals located in the Northwestern and Central Federal Districts of the Russian Federation. The majority of isolates (62/82; 75.6%) were obtained in 2021–2022. Blood was the predominant specimen type (57/82, 69.5%), whereas 21/82 isolates (25.6%) were recovered from urine or catheter-associated urine samples; the remaining isolates were obtained from respiratory specimens and purulent discharge. Hospitals 3 and 11 accounted for the largest proportions of the collection, contributing 42.7% and 18.3% of all isolates, respectively. Of the 80 patients, 48 were SARS-CoV-2-positive, 26 were SARS-CoV-2-negative, and COVID-19 status was unavailable for 6. SARS-CoV-2 status was recorded because a substantial proportion of the isolates were collected during the COVID-19 pandemic, when COVID-19-associated hospitalization may have influenced healthcare exposure and nosocomial transmission. This variable was included as descriptive metadata only and was not used in downstream statistical or phylogenetic analyses. Although the dataset predominantly comprised a cross-sectional collection of clinical isolates, it also included two pairs of sequential isolates obtained from the same patients (C57-E20/C125-E20 and C56-E20/C131-E20). Detailed metadata for all isolates are provided in **Table 1**. To preserve institutional anonymity, individual hospitals are denoted by arbitrary numerical codes (Hospitals 1–18 for the whole-genome-sequencing cohort; the targeted-sequencing cohort additionally included Hospital 19) that do not correspond to any official designation, registration number, or ranking. The same code is used consistently for a given institution throughout the text, tables, and figures.

**Table 1:** Clinical and epidemiological characteristics of the 82 C*. auris* isolates included in whole-genome sequencing.

Isolate	Isolation date	Sample type	Hospital	COVID-19
C1-E20	15.05.2017	blood	9	NO
C2-E20	08.06.2018	urine (kateter)	10	ND
C3-E20	09.06.2018	urine (kateter)	10	ND
C4-E20	10.06.2018	urine (kateter)	10	ND
C6-E20	28.03.2019	urine	8	NO
C8-E20	14.06.2019	blood	8	NO
C29-E20	08.10.2020	urine (kateter)	10	NO
C30-E20	08.10.2020	blood	10	NO
C40-E20	29.12.2020	blood	3	NO
C41-E20	29.12.2020	BAL	3	YES
C42-E20	29.12.2020	blood	3	YES
C49-E20	29.12.2020	blood	3	YES
C56-E20	14.01.2021	urine (kateter)	3	YES
C57-E20	14.01.2021	urine	3	YES
C65-E20	02.02.2021	urine	5	YES
C67-E20	13.04.2021	urine	5	ND
C69-E20	31.03.2021	blood	7	NO
C70-E20	17.02.2021	blood	6	NO
C92-E20	15.11.2020	blood	3	YES
C103-E20	13.10.2020	blood	3	YES
C107-E20	18.03.2021	urine	3	YES
C108-E20	15.11.2020	blood	3	YES
C113-E20	15.09.2020	blood	3	YES
C117-E20	15.11.2020	blood	3	YES
C121-E20	15.11.2020	blood	3	YES
C125-E20 (first case C57-E20)	18.03.2021	blood	3	YES
C127-E20	18.03.2021	urine	3	YES
C131-E20 (first case C56-E20)	18.03.2021	blood	3	YES
C155-E20	18.03.2021	blood	3	YES
C168-E20	25.05.2021	blood	3	YES
C172-E20	12.07.2021	urine	4	YES
C175-E20	16.07.2021	urine	3	YES
C182-E20	11.08.2021	blood	3	YES
C193-E20	11.08.2021	blood	3	YES
C199-E20	11.08.2021	blood	3	YES
C202-E20	01.10.2021	blood	3	NO
C210-E20	22.09.2021	blood	3	YES
C222-E20	20.10.2021	blood	3	YES
C225-E20	11.11.2021	blood	3	YES
C227-E20	02.11.2021	urine	3	YES
C230-E20	03.08.2021	urine	1	YES
C234-E20	10.11.2021	urine	1	YES
C237-E20	29.06.2021	blood	1	YES
C240-E20	17.09.2021	sputum	1	YES
C241-E20	30.07.2021	urine	1	YES
C245-E20	11.11.2021	blood	13	NO
C248-E20	08.11.2021	blood	12	NO
C257-E20	10.08.2021	blood	4	YES
C262-E20	21.02.2022	blood	2	NO
C264-E20	18.01.2022	sputum	3	YES
C265-E20	11.02.2022	blood	3	NO
C273-E20	21.02.2022	blood	3	YES
C274-Е20	27.12.2021	blood	12	NO
C275-E20	18.03.2022	pus	4	NO
C277-E20	30.03.2022	urine	14	NO
C279-E20	21.03.2022	blood	2	NO
C284-E20	17.01.2022	blood	11	NO
C285-E20	07.02.2022	blood	11	YES
C287-E20	07.02.2022	blood	11	YES
C289-E20	20.12.2021	blood	11	YES
C294-E20	08.11.2021	blood	11	YES
C300-E20	17.01.2022	blood	11	NO
C302-E20	13.04.2022	blood	11	YES
C305-E20	01.03.2022	blood	11	YES
C310-E20	14.03.2022	blood	11	NO
C315-E20	03.03.2022	blood	11	NO
C317-E20	16.03.2022	blood	11	YES
C318-E20	16.03.2022	blood	11	YES
C319-E20	10.04.2022	blood	11	YES
C320-E20	05.03.2022	blood	11	YES
C321-E20	25.03.2022	blood	11	YES
C349-E20	11.06.2021	blood	3	YES
C351-E20	25.06.2021	blood	3	YES
C356-E20	14.06.2022	urine	15	NO
C359-E20	16.04.2021	blood	3	YES
C390-E20	06.09.2022	blood	3	NO
C399-E20	16.08.2022	blood	1	NO
C410-E20	10.10.2022	urine	16	NO
C457-E20	09.09.2022	blood	17	ND
C512-E20	22.12.2021	blood	3	YES
C536-E20	26.03.2023	blood	12	NO
C537-E20	23.03.2023	urine	18	ND

An additional cohort of 15 clinical *C. auris* isolates was included exclusively for targeted sequencing of the *FCY2* gene to extend the assessment of mutations associated with elevated 5-flucytosine MICs (≥128 mg/L). This cohort comprised one isolate recovered from a urine specimen at Hospital 10 in 2022 (C374-E20), 13 isolates collected from Hospital 17 between March and June 2024, and one isolate obtained from Hospital 19 in June 2024 (C697-E20). These isolates were not subjected to whole-genome sequencing and were therefore excluded from the population structure and phylogenetic analyses. Detailed metadata for this subset are provided in **Supplementary Table 1**. For primary identification, re-identification, and all experiments described in this study, isolates were subcultured on Sabouraud dextrose agar supplemented with chloramphenicol (Conda Laboratories, Madrid, Spain) and incubated at 37 °C for 18–24 h. Fresh overnight cultures were used for antifungal susceptibility testing and molecular genetic analyses.

### 2.2 Susceptibility of *C. auris* to antifungal drugs

Minimum inhibitory concentrations (MICs, mg/L) of antifungal agents were determined by the broth microdilution method based on the EUCAST E.Def 7.4 reference protocol. Susceptibility testing was performed in RPMI-1640 medium with L-glutamine, without bicarbonate, supplemented with 2% D-glucose, and buffered to pH 7.0 with 0.165 M 3-(N-morpholino)propanesulfonic acid (MOPS). The final inoculum density was 0.5–2.5 × 10^5^ CFU/mL. Microdilution was performed in flat-bottom 96-well plates, which were incubated at 35 °C for 24 h. The final concentration ranges of the twofold serial dilutions were as follows: amphotericin B — 0.016–16 mg/L, fluconazole — 0.125–64 mg/L, itraconazole, voriconazole, and posaconazole — 0.008–8 mg/L, anidulafungin, micafungin, and caspofungin — 0.008–8 mg/L, and 5-flucytosine — 0.06–128 mg/L. Although EUCAST does not currently recommend caspofungin MIC determination for clinical interpretation of *C. auris* susceptibility due to substantial inter-laboratory variability, and recommends anidulafungin or micafungin as surrogate markers for echinocandin susceptibility, caspofungin was included in the present study for comparative purposes alongside the other echinocandins. The 5-flucytosine range was extended above the EUCAST upper limit to allow accurate quantification of MICs for isolates with high-level resistance. *Candida parapsilosis* ATCC 22019 was used as the quality control strain, and MICs for the QC strain remained within the EUCAST-recommended ranges throughout the study. MICs were determined spectrophotometrically by measuring optical density at 530 nm in a Multiskan GO microplate reader (Thermo Fisher Scientific Oy, Finland). For amphotericin B, the MIC was defined as the lowest drug concentration resulting in at least 90% growth inhibition compared with the drug-free growth control, whereas for 5-flucytosine, azoles, and echinocandins, the MIC was defined as the lowest concentration producing at least 50% growth inhibition. Although EUCAST has recently established an epidemiological cut-off value (ECOFF) of 0.5 mg/L for 5-flucytosine in *C. auris* (Arendrup et al., 2026), clinical breakpoints have not yet been defined; therefore, isolates were not categorized as susceptible, intermediate, or resistant (S/I/R).

### 2.3 DNA extraction

Genomic DNA was extracted from overnight cultures of *C. auris* using a modified CTAB/chloroform–isoamyl alcohol protocol, adapted for fungal cells based on methods described for fungal DNA extraction (Möller et al., 1992). Prior to extraction, cell suspensions were prepared in physiological saline and homogenized using a tissue homogenizer to ensure efficient cell disruption.

### 2.4 Assessment of DNA quantity and quality

DNA concentration was measured on a Qubit 4.0 fluorometer using the Qubit™ dsDNA HS Assay Kit (both from Thermo Fisher Scientific, Waltham, MA, USA), according to the manufacturer’s instructions. The working concentration of DNA samples obtained from *C. auris* cultures was adjusted to 10 ng/μL by dilution.

DNA purity was assessed by UV spectrophotometry on a Nano-300 instrument (Allsheng, Hangzhou, China) by measuring absorbance at 230, 260, and 280 nm.

### 2.5 Targeted sequencing of the ITS rDNA region

Molecular identification was performed by targeted sequencing of the complete internal transcribed spacer (ITS) region, spanning ITS1, the 5.8S rDNA and ITS2, of the ribosomal DNA (rDNA) in accordance with the CLSI MM18 (Clinical and Laboratory Standards Institute, 2018). Amplification of the ITS region was carried out using universal fungal primers ITS5 (5′-GGAAGTAAAAGTCGTAACAAGG-3′) and ITS4 (5′-TCCTCCGCTTATTGATATGC-3′) (White et al., 1990). PCR amplicon quality was assessed by electrophoresis in 1.5% agarose gel in TBE buffer containing ethidium bromide, with visualization under UV light.

Sanger sequencing was performed on an ABI 3500 Genetic Analyzer (Applied Biosystems, Foster City, CA, USA) using the BigDye Terminator v3.1 Cycle Sequencing Kit (Thermo Fisher Scientific, Waltham, MA, USA) in a 10 μL reaction volume, according to the manufacturer’s instructions. Unincorporated terminators were removed using the BigDye XTerminator Purification Kit (Thermo Fisher Scientific, Waltham, MA, USA). Species identification was determined by aligning the resulting nucleotide sequences against the GenBank reference database using BLASTn.

### 2.6 Library preparation and NGS sequencing

Sequencing libraries were prepared from genomic DNA using the Illumina DNA Prep (M) Tagmentation kit with IDT for Illumina DNA/RNA UD Indexes Set A (Illumina, San Diego, CA, USA), according to the manufacturer’s protocol. The pooled library was denatured and diluted to a final loading concentration of 1.3 pM prior to sequencing. A PhiX control library (Illumina, San Diego, CA, USA) was spiked into the pool at 1% to monitor sequencing performance. Paired-end sequencing (2 × 150 bp) was performed on an Illumina NextSeq 550 platform using a NextSeq 500/550 High Output Kit v2.5 (300 cycles) (Illumina, San Diego, CA, USA).

### 2.7 Quality control of sequencing reads

Raw read quality was assessed with FastQC (v0.12.1) (Andrews, 2010), and summaries were visualized with MultiQC (Ewels et al., 2016). Adapter sequences and low-quality bases were trimmed with fastp (v0.23.4) (Chen et al., 2018).

### 2.8 Read alignment and variant calling

Cleaned paired-end reads were aligned to the *C. auris* reference genome strain B8441 (GenBank accession: GCA_002759435.3) using BWA-MEM (v0.7.17) (Li, 2013). Duplicate reads were marked with Picard MarkDuplicates (v3.1.1). Variant calling was performed using GATK HaplotypeCaller (4.6.1.0) in GVCF mode with ploidy 1 for haploids. Individual GVCFs were combined using GATK CombineGVCFs, followed by joint genotyping with GATK GenotypeGVCFs. Variant filtering was conducted according to the GATK best practices for hard filtering. Single nucleotide variants (SNVs) and insertions/deletions (indels) were filtered separately using GATK SelectVariants and GATK VariantFiltration with the following parameters: SNVs: DP < 10, QD < 2.0, QUAL < 30.0, SOR > 3.0, FS > 60.0, MQ < 40.0, MQRankSum < -12.5, ReadPosRankSum < -8.0. Indels: DP < 10, QD < 2.0, QUAL < 30.0, FS > 200.0, ReadPosRankSum < -20.0. Additional filters were applied using bcftools (v1.22) (Danecek et al., 2021): genotype quality (GQ) ≥ 80 and an allele depth (AD) ≥ 5 for the alternative allele(s). Annotation of variants and prediction of their functional impact on genes and proteins were carried out using Ensembl Variant Effect Predictor (VEP).

### 2.9 Time-calibrated phylogenetic analysis

The VCF file containing only single nucleotide polymorphisms (SNPs) was converted into a multiple sequence alignment using vcf2phylip (v2.9) (Ortiz, 2019). To infer a maximum-likelihood tree from the SNP alignment and estimate divergence times among lineages IQ-TREE (v.3.0.1) (Minh et al., 2020) was used with the following parameters: ModelFinder (-m MFP) for model selection, 1000 ultrafast bootstrap replicates (-B 1000), and SH-aLRT branch support (-alrt 1000). Additionally, Bayesian phylogenetic dating was performed with BEAST 2 (v2.7.7). We tested combinations of substitution models (GTR and HKY), clock models (strict and OptimisedRelaxedClock), and tree priors (Yule process and Coalescent Exponential Growth). For each model combination, the MCMC chain was run for 50 000 000 iterations, sampling every 1 000 states, with the first 10 % of trees discarded as burn-in. Convergence was assessed using Effective Sample Size (ESS) in Tracer v1.7.2. The best-fitting model was GTR with a strict clock and a Coalescent Exponential Growth prior, which yielded all ESS values exceeding 1 300. The maximum-clade-credibility tree was summarized with TreeAnnotator after discarding the first 10% of trees as burn-in.

### 2.10 Integration of public short-read data

To improve phylogenetic resolution, short-read whole-genome sequencing (WGS) data from 341 additional *C. auris* isolates spanning multiple clades and subclades were downloaded from the NCBI Sequence Read Archive (SRA) (see Supplementary Table “**Supplementary Table 2**_341ncbi strains “). These samples were processed using the same alignment and variant-calling pipeline as the 82 isolates described above, with additional filtering to retain only SNPs present in at least 90% of the samples. The resulting VCF files were converted to multiple sequence alignments with vcf2phylip and used to infer maximum-likelihood phylogenies with IQ-TREE (v.3.0.1) under the same parameters.

### 2.11 Tree visualization

Phylogenetic trees were visualized using Interactive Tree of Life (iTOL) (Letunic and Bork, 2024), and TreeViewer (Bianchini and Sánchez‐Baracaldo, 2024).

### 2.12 Pairwise SNP distance matrix

A symmetrical pairwise SNP distance matrix was computed using psdm (v0.3.0) (Hall, 2021), which calculates the number of differing nucleotide positions between each pair of isolates while ignoring positions containing ambiguous bases (N, -). The resulting matrix provided the raw pairwise distances for all subsequent analyses.

For each isolate, a mean pairwise SNP distance was derived as the arithmetic mean of its pairwise distances to all other isolates belonging to the same predefined group (e.g., city or hospital). This metric summarizes the average genomic divergence of an isolate from its group members and was used to quantify within-group genetic diversity. Distributions of mean pairwise SNP distances were visualized using boxplots with superimposed stripplots to show individual isolate values.

To evaluate inter-hospital transmission patterns, we compared the distributions of raw pairwise SNP distances for isolate pairs originating from the same hospital (“within” category) versus those from different hospitals (“between” category). A pair was assigned to the “within hospital” category if both isolates were recovered from the same healthcare facility, and to the “between hospitals” category if they originated from different facilities. We then calculated summary statistics (median, mean, standard deviation, minimum, and maximum) for each group. The distributions of within-hospital and between-hospital distances were visualized as side-by-side boxplots, where each point represents the distance of a single isolate pair.

For the global context analysis, we selected the five countries with the lowest median pairwise SNP distance from Moscow isolates (based on all Moscow–country pairs) and included three geographically distant reference countries (USA, India, South Africa) as contrasts. Boxplots were constructed to display the pairwise SNP distance distributions between isolates from Moscow and each selected country, as well as between St. Petersburg isolates and the same set of countries.

Between-group comparisons were tested for statistical significance using the two-sided Mann–Whitney U test. For comparisons involving more than two groups, the Kruskal–Wallis test was applied, followed by Benjamini–Hochberg false discovery rate (FDR) correction.

All statistical analyses and plotting were performed in Python (v3.11) using the packages pandas, numpy, scipy, statsmodels, matplotlib, and seaborn.

### 2.13 PCR amplification of the *FCY2* gene

Targeted Sanger sequencing of the *FCY2* gene was performed for two purposes: to confirm the T1148G (L383*) mutation initially identified by whole-genome sequencing and associated with elevated 5-flucytosine MICs, and to characterize *FCY2* polymorphisms in 15 additional isolates not subjected to WGS but exhibiting high 5-flucytosine MICs (MIC ≥128 mg/L; described in the Materials and Methods («Isolates and culture conditions», **Supplementary Table 1**).

### 2.14 Primer design

Primers for amplification and sequencing of the *FCY2* gene were designed using the Primer3web v4.1.0 server (https://primer3.ut.ee/; (Untergasser et al., 2012)).

Primers were designed to be 18–22 nucleotides in length, with a melting temperature (Tm) of 58–60 °C and minimal predicted self-complementarity or primer-dimer formation. The *FCY2* gene sequence from the *C. auris* B8441 reference genome (assembly GCA_002759435.3, chromosome 7, GenBank accession CM076444.1; locus tag B9J08_05445) was used as the template for primer design. The complete list of primers used for amplification and sequencing is provided in **Supplementary Table 3** «Oligonucleotides used for sequencing of the *FCY2* gene in *C. auris».*

### 2.15 PCR product purification and sanger sequencing

PCR was performed in a final volume of 50 μL containing 5 μL of dNTP mix (2.5 mM each), 5 μL of 10× reaction buffer (750 mM Tris-HCl, pH 8.8; 200 mM (NH_4_)_2_SO_4_; 0.1% Tween 20), 2 μL of each primer (20 pmol/μL), 5 μL of 25 mM MgCl_2_, 2.5 μL of genomic DNA (10 ng/μL), 0.3 μL of Taq DNA polymerase (5 U/μL; New England Biolabs, Ipswich, MA, USA), and 28.2 μL of nuclease-free water.

Thermal cycling was performed on a CFX96 Touch Real-Time PCR Detection System (Bio-Rad Laboratories, Hercules, CA, USA), used as a conventional thermal cycler for end-point amplification, with the following program: an initial denaturation at 94 °C for 5 min, followed by 35 cycles of 94 °C for 30 s, 58.3 °C for 40 s, and 72 °C for 50 s, with a final extension at 72 °C for 10 min. PCR product size and quality were evaluated by electrophoresis in a 6% polyacrylamide gel in TBE buffer, with ethidium bromide staining and visualization on a UV transilluminator.

PCR products were purified using the GeneJET PCR Purification Kit (Thermo Fisher Scientific, Waltham, MA, USA) and used as templates for cycle sequencing with the BrilliantDye Terminator v3.1 Cycle Sequencing Kit (NimaGen, Nijmegen, the Netherlands). Sequencing was carried out using the same primers listed in **Supplementary Table 3**. Excess terminators and salts were removed using the BigDye XTerminator Purification Kit (Thermo Fisher Scientific, Waltham, MA, USA). Sequencing products were analyzed by capillary electrophoresis on an ABI 3500 Genetic Analyzer (Applied Biosystems, Foster City, CA, USA).

### 2.16 Bioinformatic analysis of the *FCY2* gene

Sanger chromatograms were inspected and trimmed in Variant Reporter Software v3.0 (Applied Biosystems, Foster City, CA, USA). Forward and reverse reads from each isolate were assembled into consensus sequences and aligned to the *FCY2* reference sequence (assembly GCA_002759435.3, locus tag B9J08_05445) using SeaView v4.7 (Gouy et al., 2010). Nucleotide and predicted amino acid changes relative to the reference were identified by manual inspection of the alignments and verified by re-examining the corresponding positions in the original chromatograms.

## 3 Results

### 3.1 Antifungal resistance of *C. auris*: phenotypic and genotypic insights

Antifungal susceptibility profiles of all 82 C*. auris* isolates are presented in **Table 2**. Clinical interpretation followed EUCAST breakpoints v.12.1 for amphotericin B, anidulafungin, and micafungin, and the EUCAST ECOFF for 5-flucytosine; no clinical breakpoints are currently defined for *C. auris* against caspofungin or the triazoles.

**Table 2:** Distribution of minimum inhibitory concentrations (MICs) of nine antifungal drugs for 82 clinical *C. auris* isolates, determined by EUCAST E.Def 7.4 broth microdilution.

Antifungal	MIC range (mg/L)	MIC_50_ (mg/L)	MIC_90_ (mg/L)	Number of isolates with MIC (mg/L) of:											
				128	64	32	16	8	4	2	1	0.5	0.25	0.125	≤0.06
Amphotericin B	0.25–4	1	2						1	9	44	27	1		
Voriconazole	0.015–4	2	2						8	44	12	7		4	7
Itraconazole	0.015–0.5	0.125	0.25									4	23	40	15
Posaconazole	0.008–0.125	0.125	0.125											68	14
Fluconazole	8–64	64	64		71	3	6	2							
Anidulafungin	0.015–0.25	0.06	0.125										3	28	51
Caspofungin	0.03–0.25	0.125	0.125										3	57	22
Micafungin	0.03–0.125	0.06	0.125											34	48
5-Flucytosine	0.06–128	0.06	0.125	3									1	16	62

Elevated fluconazole MICs were a defining feature of the cohort: MICs were uniformly elevated, with 80 of 82 isolates (97.6%) showing MICs ≥16 mg/L and the modal MIC at the upper limit of the tested range. The remaining triazoles displayed a markedly different pattern. Voriconazole MICs were broadly distributed, while itraconazole and posaconazole MICs were narrowly distributed with low modal values (**Table 2**). Activity of amphotericin B was preserved in the vast majority of isolates, with 81 of 82 (98.8%) falling within the wild-type distribution. A single outlier (C321-E20) exceeded the EUCAST resistance breakpoint and was categorized as resistant (**Supplementary Table 4** «Minimum inhibitory concentrations (MIC, mg/L) of nine antifungal agents for clinical isolates of *C. auris*»).

Echinocandins retained full *in vitro* activity: all 82 isolates (100%) were susceptible to anidulafungin and micafungin by EUCAST criteria. Caspofungin MICs were similarly low and homogeneously distributed (**Table 2**). The most notable finding concerned 5-flucytosine. While most isolates fell within the wild-type distribution, three (C245-E20, C285-E20, and C310-E20; 3.7%) exhibited MICs of 128 mg/L — more than 250-fold above the ECOFF — and were classified as non-wild-type (**Supplementary Table 4** «Minimum inhibitory concentrations (MIC, mg/L) of nine antifungal agents for clinical isolates of *C. auris*»).

**Figure 1:**
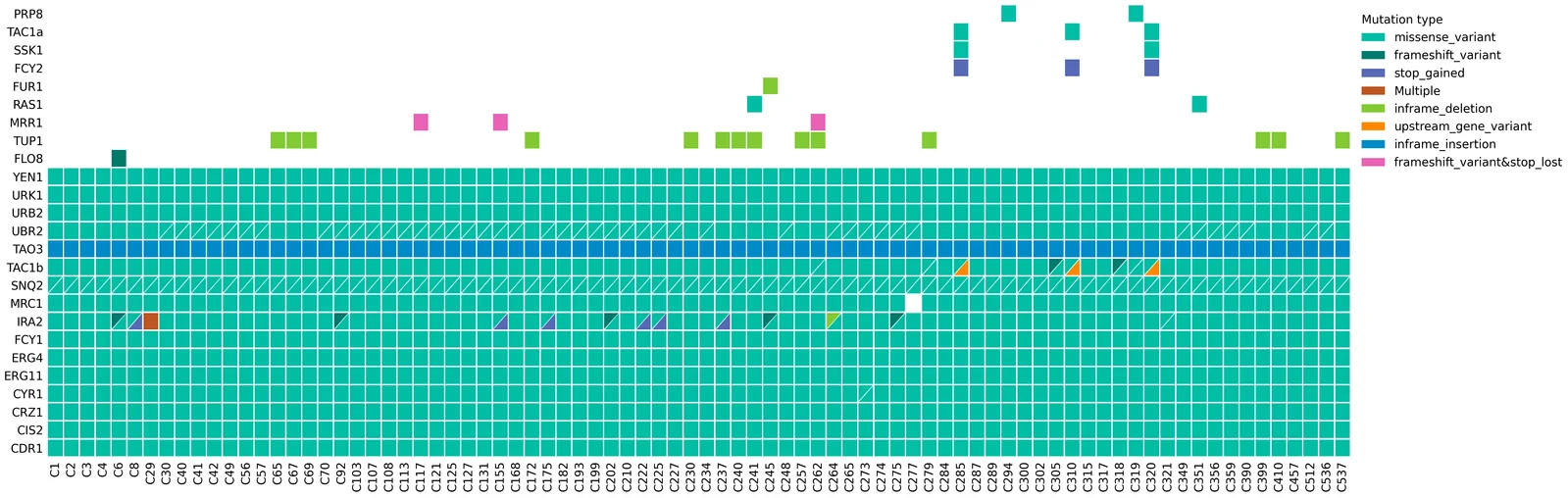
Overview of principal variants in antifungal resistance candidate genes among 82 C*. auris* isolates from the Russian Federation.Rows correspond to selected genes from the 74-gene antifungal resistance candidate panel, and columns correspond to individual isolates. Colors denote the predicted consequence of each variant. The full variant dataset, including synonymous substitutions and variants in all candidate genes, is provided in **Supplementary Table 6**.

In the course of genome analysis of 82 C*. auris* isolates collected in the Russian Federation, single nucleotide variants (SNVs), insertions, and deletions were identified in genes potentially associated with antifungal resistance. A panel of 74 candidate genes previously implicated in antifungal resistance was compiled through a literature review (**Supplementary Table 5** – Genes associated with antifungal resistance”). All variants identified within these genes across the 82 isolates, including synonymous and noncoding substitutions, are listed in **Supplementary Table 6**. An overview of the principal variants characterizing the studied cohort is shown in **Figure 1**.

All 82 isolates carried the K143R substitution in *ERG11*. The two isolates with the lowest fluconazole MICs in the cohort (8 mg/L; C399-E20 and C410-E20) also carried this substitution. No additional variants were detected in *ERG11*. A non-synonymous T148I substitution in *ERG7*, encoding lanosterol synthase, an early enzyme in the ergosterol biosynthesis pathway, was identified in a single isolate (C512-E20). All isolates carried a c.576G>T (p.Met192Ile) substitution in *ERG4* and a synonymous c.870A>C (p.Thr290=) variant in *ERG5*, both encoding enzymes that catalyze the final steps of ergosterol biosynthesis. In contrast, *ERG3* and *ERG6* sequences were identical to the reference genome in all 82 isolates. Beyond the ergosterol biosynthesis pathway, variants were also identified in genes encoding ABC transporters that mediate efflux of antifungal compounds. All 82 isolates carried missense substitutions in *CDR1* (c.2110G>T, p.Val704Leu) and in *SNQ2* (A156T and K52N).

Genetic variants were also identified in transcriptional regulators of efflux pump expression. The I448S substitution in *TAC1A* was detected in three isolates (C285-E20, C310-E20, and C320-E20), all recovered from the same healthcare facility. All isolates carried an A640V substitution in *TAC1B*, along with several additional substitutions in a subset of strains; none of these variants showed an association with elevated amphotericin B MICs. Three other strains (C117-E20, C155-E20, and C262-E20) carried a stop-lost variant (p.Ter907Tyrext*) in *MRR1*, a gene involved in the regulation of drug resistance-associated transporters. In addition to known transcriptional regulators such as *TAC1A* and *MRR1*, a deletion in the *TUP1* gene (B9J08_03515) was identified in 16 isolates. *TUP1* encodes a general transcriptional corepressor involved in regulating a wide range of cellular processes. Among the 16 isolates carrying this deletion, 14 (87.5%) exhibited voriconazole MICs ≤0.5 mg/L.

Beyond efflux regulators, two additional substitutions were present in all 82 isolates: S237Y in *CRZ1* and the synonymous S436S in *CAS5*. An F1148I substitution in *IRA2*, encoding a regulator of the Ras/cAMP signaling pathway, was present in all 82 isolates, along with several low-frequency variants in the same gene. The replication checkpoint mediator *MRC1* carried a Q313R change (81/82; 98.8%). In the ubiquitin-mediated protein degradation system, *UBR2* harbored an A316T substitution detected in 100% of isolates (82/82), as well as an intermediate-frequency F160L polymorphism (n = 44).

Alongside the analysis of azole-associated resistance genes, mutations were investigated in genes potentially influencing susceptibility to amphotericin B. All 82 isolates carry the synonymous G672T (A224A) variant in *BRE1*. In addition to this conserved variant, a missense K719N substitution in *STE6*, encoding an ABC transporter involved in pheromone export, was uniformly present across the cohort. Apart from these cohort-wide variants, a strain-specific A164V substitution was identified in *ARG1*, encoding argininosuccinate synthase, in a single isolate (C356-E20).

Additionally, a small number of strain-specific substitutions were identified in regulators of signaling and morphogenesis, including *RAS1* (in C241-E20 and C351-E20), *CYR1* (C273-E20), *SSK1* (C285-E20 and C320-E20), *TAO3* (C230-E20 and C241-E20), and *FLO8* (C6-E20). Low-frequency variants were similarly observed in genes involved in vesicular trafficking and pre-mRNA splicing: *GEA2* (C262-E20 and C279-E20) and *PRP8* (C294-E20 and C319-E20). The complete list of variants and corresponding isolates is provided in **Supplementary Table 6** «Mutations in genes associated with antifungal resistance in 82 C. *auris* strains». To explore whether these rare variants might be related to antifungal susceptibility, we examined MIC profiles in relation to variant carriage across the candidate loci listed above (**Supplementary Table 6**). Because most variants were detected in only one or two isolates and suitable comparison groups were unavailable, formal genotype–phenotype association testing was not feasible. No consistent pattern of elevated MIC values was observed among isolates carrying these variants.

Consistent with the uniformly low echinocandin MICs (see the antifungal-susceptibility results above), no variants were detected in *FKS1* or *FKS2*, including the hot-spot regions associated with echinocandin resistance. All 82 isolates carried a K74E substitution in *CIS2*, encoding a protein involved in lipid metabolism and membrane integrity, as well as identical variants in *URK1*, *URB2*, and *YEN1*, the functional relevance of which to antifungal susceptibility remains unclear.

We examined genetic determinants of 5-flucytosine susceptibility, focusing on *FCY1*, *FCY2*, and *FUR1*, which encode key components of the cytosine permease and pyrimidine salvage pathway in *Candida* species. Among the 82 isolates, 78 (95.1%) exhibited 5-flucytosine MICs ≤0.125 mg/L, a single isolate (C320-E20) showed an MIC of 0.25 mg/L — above the cohort modal range but within the EUCAST wild-type distribution — and three isolates (C245-E20, C285-E20, and C310-E20) displayed markedly elevated 5-flucytosine MICs of 128 mg/L (non-wild-type by the EUCAST ECOFF).

WGS analysis identified two distinct molecular mechanisms underlying these elevated MICs. In *FCY2*, encoding the cytosine permease, a T1148G substitution introducing a premature stop codon (L383*) was detected in three isolates: C285-E20 and C310-E20, both with 5-flucytosine MICs of 128 mg/L, and C320-E20, which carried the same truncating variant despite displaying an MIC of only 0.25 mg/L (within the EUCAST wild-type distribution). The third high-level resistant isolate, C245-E20 (MIC 128 mg/L), did not carry the *FCY2* L383* variant but instead harbored a deletion in *FUR1*, encoding uracil phosphoribosyltransferase. An S70R substitution in *FCY1*, encoding cytosine deaminase, was detected in all 82 isolates and is therefore unlikely to contribute to differential 5-flucytosine susceptibility within the cohort.

Building on these WGS findings, we developed a Sanger-based targeted sequencing assay for *FCY2* and applied it to an additional collection of 15 C*. auris* isolates that exhibited 5-flucytosine MICs of 128 mg/L but had not been included in the WGS analysis. To validate the assay, three WGS-characterized isolates carrying the L383* variant (C285-E20, C310-E20, and C320-E20) were re-sequenced, and the mutation was confirmed in all three. C321-E20 (MIC 0.06 mg/L), which lacked the variant by WGS, was included as a negative control and was confirmed wild-type by Sanger sequencing. Among the 15 additional isolates with elevated 5-flucytosine MICs, 14 carried the identical T1148G (L383*) variant in *FCY2*, while one isolate (C697-E20) harbored a previously undescribed C983A substitution introducing a second premature stop codon (S328*).

### 3.2 Phylogenetic analysis and clade classification

Phylogenetic analysis based on a SNP alignment of all 82 Russian isolates together with 341 publicly available global *C. auris* genomes representing the recognized clades placed all Russian isolates within clade I (the South Asian clade; **Supplementary Figure 1**). The Russian isolates formed a single, well-supported monophyletic cluster, nested among clade I isolates of Italian and US origin (**Supplementary Figure 2**). This topology suggests limited introduction events of clade I *C. auris* into Russia, followed by local divergence, rather than through repeated independent introductions from divergent international sub-lineages.

**Figure 2:**
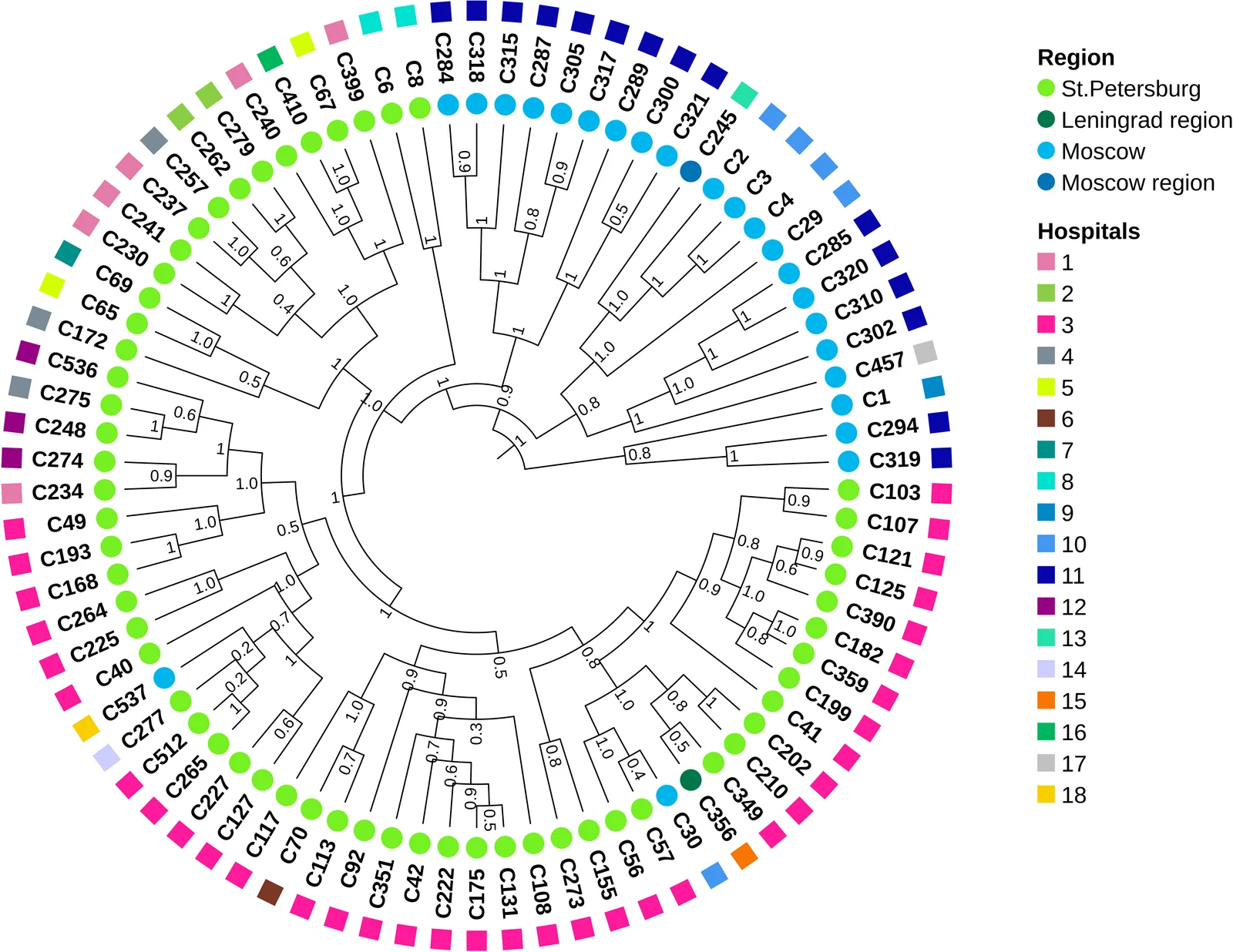
Bayesian phylogenetic tree of 82 Russian *C. auris* isolates inferred under the GTR substitution model in BEAST. Posterior probabilities are shown at internal nodes. Tip colors indicate region of isolation (light green - Saint Petersburg; dark green - Leningrad oblast; light blue - Moscow; dark blue - Moscow oblast).

The Russia-only phylogenetic analysis revealed a strongly structured topology with two contrasting patterns of geographic clustering (**Figure 2**). All 57 Saint Petersburg isolates were contained within a single monophyletic clade, together with the single Leningrad oblast isolate (C356-E20) and two Moscow isolates (C30-E20 and C537-E20). Moscow isolates, by contrast, did not form a single monophyletic group: they were paraphyletically distributed across multiple distinct lineages — including one large subclade dominated by a single hospital and several smaller, more divergent lineages — together with the single isolate from Moscow oblast (C245-E20). Thus, the topology shows a single dominant lineage circulating in Saint Petersburg alongside multiple co-existing lineages in Moscow.

**Figure 3:**
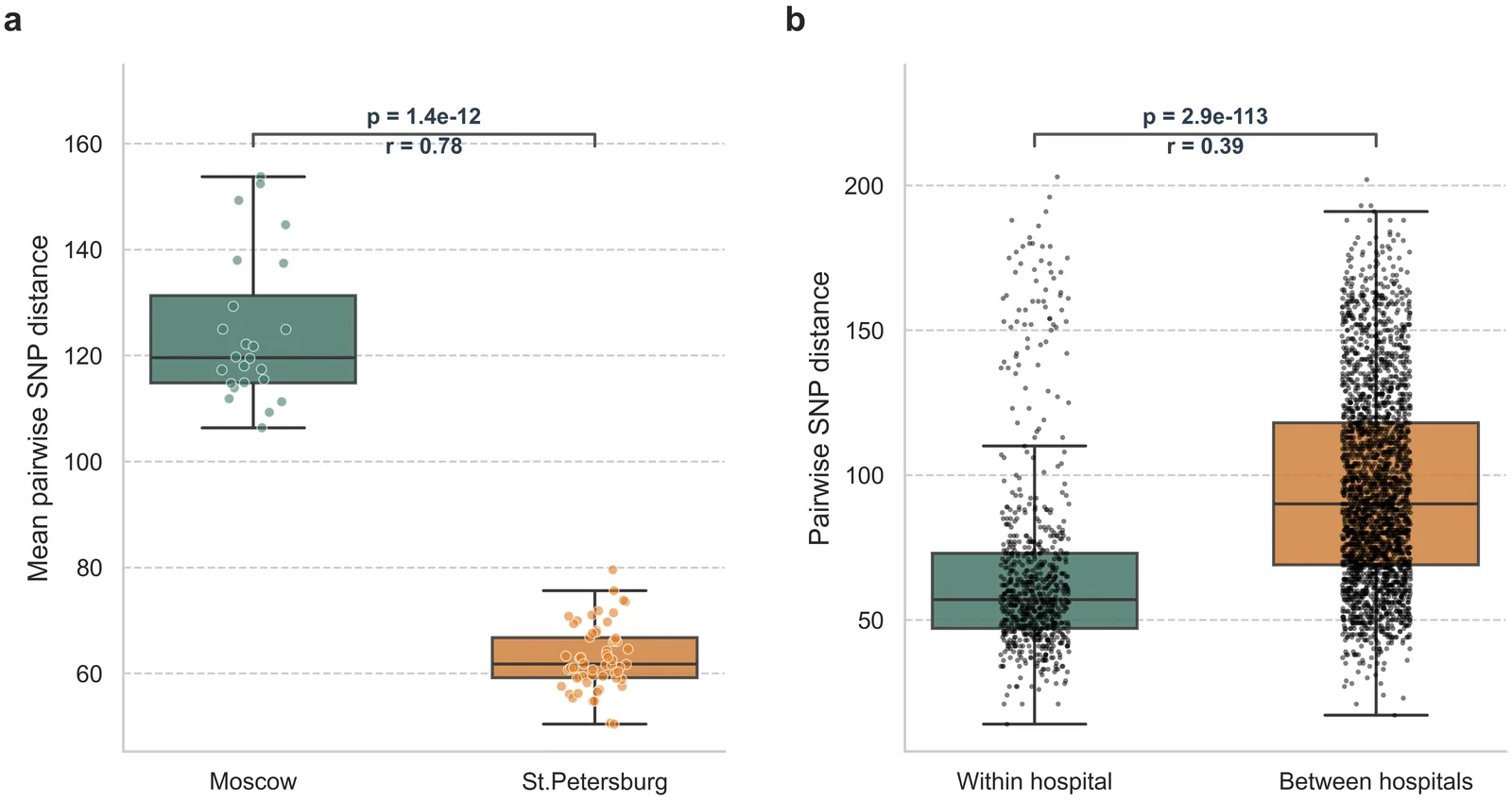
Genomic diversity of *C. auris* isolates from Russia. **(a)** Distribution of per-isolate mean pairwise SNP distances for isolates from Moscow (n = 24) and St. Petersburg (n = 58). Each point represents the mean pairwise SNP distance of a single isolate to all other isolates from the same city (i.e., the average genomic divergence of that isolate from its regional group). Boxplots show the median (central horizontal line), interquartile range (IQR; box), and whiskers extending to the most extreme values ​​within 1.5 × IQR. Individual observations are overlaid as points.**(b)** Distribution of raw pairwise SNP distances for isolate pairs originating from the same hospital (“Within hospital”) and from different hospitals (“Between hospitals”). Each point represents the SNP distance for a single pair of isolates. Boxplots show the median (central horizontal line), IQR (box), and whiskers extending to the most extreme values ​​within 1.5 × IQR; individual distances are overlaid as a strip plot. For both comparisons, the Mann–Whitney U test p-value and the corresponding effect size (r) are shown above the plots.

The Saint Petersburg macrocluster was larger but, on a per-pair basis, less internally diverse than the Moscow macrocluster (median pairwise SNP distance: 61.8 vs 119.6 SNP in Moscow; Mann–Whitney U test, p = 1.4 × 10^-12^, **Figure 3a**). The effect size, quantified as r = Z/√N, was 0.78, indicating a large effect. In addition, raw pairwise SNP distances between isolates recovered from the same hospital were significantly lower than those between isolates from different hospitals (median 57 vs 90 SNP; Mann–Whitney U test, p = 2.9 × 10^-113^, **Figure 3b**) with an effect size of r = 0.39, corresponding to a moderate effect. This Saint Petersburg–dominated monophyletic clade comprised 60 isolates (57 from Saint Petersburg, one from Leningrad oblast, and two from Moscow) recovered from 11 healthcare institutions. Several well-supported subclusters were identified within this lineage, and isolates collected in different years often remained closely related phylogenetically. In addition, isolates from multiple Saint Petersburg institutions were interspersed within the same broader phylogenetic structure, indicating that the observed diversity was not confined to single hospitals.

The Moscow macrocluster was smaller and topologically more compact, comprising 22 isolates from seven institutions. Its core was formed by a dense and genetically homogeneous subcluster centered on Hospital 11, which contributed the largest share of Moscow isolates. Compared with the Saint Petersburg lineage, the Moscow cluster showed less internal branching on the tree (owing to its smaller size) yet a higher per-pair genomic diversity.

The single isolate from Moscow Oblast fell within the Moscow cluster, and the single isolate from Leningrad Oblast clustered within the Saint Petersburg lineage. These observations do not support the existence of independent peripheral populations genetically distinct from the metropolitan reservoirs. Instead, this pattern suggests that regional institutions are epidemiologically connected to the major urban centers and participate in the same broader transmission networks.

The two pairs of sequential isolates recovered from the same patients (C57-E20/C125-E20 and C56-E20/C131-E20) provide further insight into transmission dynamics. Rather than forming two distinct patient-specific terminal branches, all four isolates were embedded within the broader Saint Petersburg hospital-associated lineage. Moreover, the later bloodstream isolates were not placed as exclusive sister taxa to the earlier urinary isolates, but instead fell elsewhere within the same cluster. This pattern suggests that repeated isolation from the same patient does not represent an epidemiologically independent event disconnected from the surrounding transmission context. Instead, the data are consistent with within-host persistence occurring in the setting of ongoing circulation of closely related genotypes in the hospital environment, making it difficult to distinguish microevolution, reacquisition, and repeated exposure to the same institutional reservoir on phylogenetic grounds alone.

**Figure 4:**
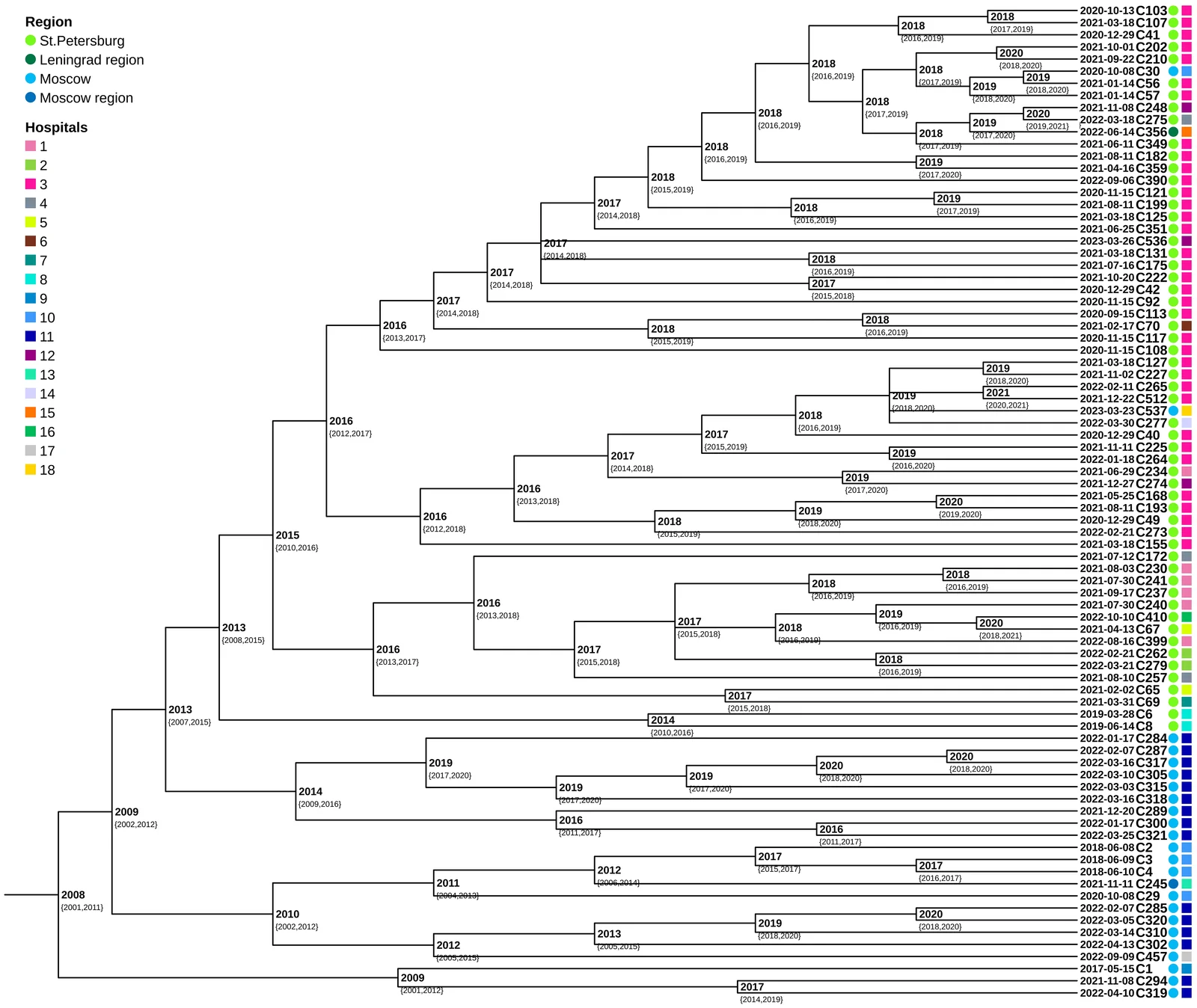
Time-calibrated phylogeny of 82 Russian *C. auris* isolates. The tree was inferred in IQ-TREE with tip dates corresponding to the isolation date of each isolate. Internal node labels indicate inferred dates of the most recent common ancestors. Tip colors indicate region of isolation (light green - Saint Petersburg; dark green - Leningrad oblast; light blue - Moscow; dark blue - Moscow oblast); colored squares denote the originating healthcare institution (Hospitals 1–18). Tip dates correspond to dates of isolate collection. Isolate identifiers correspond to those listed in **Table 1**.

To add a temporal dimension to the phylogenetic structure described above, we reconstructed a time-calibrated tree. This analysis placed the most recent common ancestor of the sampled Russian isolates at approximately 2008 and the major internal node giving rise to most currently sampled Russian lineages at around 2014, both predating the first laboratory-confirmed Russian case in 2017. Thus, the dated phylogeny indicates that the lineage represented in the present collection had already split into multiple descendant lineages before its clinical recognition. Consistent with the previously observed topology, isolates collected in 2021–2023 were distributed across multiple internal branches rather than forming a single recent terminal expansion, supporting the persistence of several related lineages over time (**Figure 4**).

## 4 Discussion

### 4.1 Genetic determinants of antifungal resistance in *C. auris*

#### 4.1.1 Mechanisms of azole resistance in *C.auris*

Azole resistance in *C. auris* classically originates from amino-acid substitutions in *ERG11* (Y132F, K143R, F126L, and others) that reduce the affinity of lanosterol 14α-demethylase for triazoles (AlJindan et al., 2020; Rybak et al., 2021). Target-site changes alone, however, rarely account for the full resistant phenotype and typically operate together with at least two additional regulatory layers. The first is transcriptional reprogramming of the sterol pathway by *UPC2*, which can be activated either by gain-of-function mutations or by stress-induced upregulation. Active *UPC2* increases the expression of *ERG11* and other ergosterol-biosynthesis genes (*ERG1*, *ERG2*, *ERG3*, *ERG5*, *ERG6*, *ERG24*–*ERG28*), as well as early enzymes of the mevalonate branch, partially compensating for target inhibition and sterol imbalance (Li et al., 2024b). The second is induction of drug efflux, primarily through *CDR1* under the control of *TAC1B* (and, to a lesser extent, *TAC1A*), which actively extrudes azoles from the cell. When target-site substitutions, sterol-pathway adaptation, and efflux induction co-occur, the result is high-level azole resistance (Li et al., 2021; Barker et al., 2025).

In our dataset, fluconazole MICs were uniformly elevated: 80 of 82 isolates (97.6%) showed MICs ≥16 mg/L, and only two isolates (C399-E20 and C410-E20) had MICs of 8 mg/L. All isolates harbored the K143R substitution in *ERG11*, which is characteristic of the South Asian clade (Clade I) and is often cited as a lineage-specific marker of azole resistance (in some articles, it co-occurs with Y132F) (Healey et al., 2018; Lee et al., 2023). All isolates also carried *TAC1B* A640V, a canonical gain-of-function mutation that increases resistance to fluconazole by activating drug efflux, primarily via *CDR1*. Finally, all isolates carried *CDR1* V704L, a variant repeatedly detected in clusters of Clade I outbreaks (e.g., in New York) and in other settings, where it accompanies *ERG11* K143R and high-level azole resistance (Jacobs et al., 2022; Castanheira et al., 2024). The combined occurrence of target-site (*ERG11* K143R) and efflux-related (*TAC1B* A640V; *CDR1* V704L) alterations in every isolate provides a plausible mechanistic basis for the resistance phenotype observed. *SNQ2* encodes an ABC transporter implicated in efflux of azoles, and its transcription is co-induced with *CDR1* in mature *C. auris* biofilms, contributing to the reduced azole susceptibility observed in this growth state (Kean et al., 2018). In our cohort, *SNQ2* harbored two missense substitutions—A156T and K52N—present in all isolates. The K52N substitution has also been documented in all clade I isolates from a Qatari outbreak collection, which additionally carried E1464K rather than the A156T variant detected in our cohort (Ben Abid et al., 2023).The functional consequences of either substitution have not been established, and we regard them as cohort-defining variants of uncertain significance.

In contrast to the uniform alterations in *TAC1B* and *CDR1*, variants in the *MRR1*–*MDR1* pathway were restricted to a small subset of isolates. A stop-lost variant in *MRR1* (p.Ter907Tyrext*) was detected in three isolates (C117-E20, C155-E20, and C262-E20), while the remaining 79 isolates carried the wild-type allele. The fluconazole MICs of these three isolates (64 mg/L) were indistinguishable from those of the wider cohort, suggesting that this variant does not contribute to the resistance phenotype observed. Mechanistically, the variant is also distinct from the clade III *MRR1* N647T gain-of-function substitution, which constitutively upregulates *MDR1* expression and elevates fluconazole and voriconazole MICs (Li et al., 2022) rather than substituting an active-site residue, the variant identified here abolishes the native termination codon and extends the C-terminus by several amino acids, an alteration unlikely to recapitulate a gain-of-function effect.

Besides the primary azole-resistance determinants, several stress-response pathways modulate the magnitude of the resistant phenotype. The calcineurin pathway, in which the Cna1/Cnb1 phosphatase activates the transcription factor Crz1, maintains membrane and cell-wall homeostasis during azole exposure; genetic inactivation of *CNA1*, *CNB1*, or *CRZ1* in *C. auris* reduces tolerance and increases susceptibility to azoles (Cha et al., 2025). In clinical collections, recurrent driver mutations in *CRZ1* that would unambiguously explain azole resistance have not yet been described. However, in our cohort all isolates carried the amino-acid substitution *CRZ1* (S237Y), which we currently regard as a population variant.

The Ras1–Cyr1–PKA cascade provides an additional stress-response pathway relevant to azole tolerance. Ira2, a GTPase-activating protein, is the major negative regulator of Ras1 and limits Ras-dependent activation of the adenylyl cyclase Cyr1, thereby regulating intracellular cAMP levels and PKA activity. Both decreased and excessive activity of this pathway have been reported to reduce azole tolerance in *C. auris* under antifungal stress (Kim et al., 2021, 2023). In our cohort, all 82 isolates carried the *IRA2* F1148I substitution, while several additional rare *IRA2* variants, including frameshift and nonsense alleles, were found in individual isolates. Given that Ras–cAMP/PKA signaling supports broad stress adaptation rather than azole resistance specifically, the contribution of these variants to the observed phenotype requires direct experimental validation.

A second mechanism controlling azole tolerance operates through the ubiquitin–proteasome regulator Ubr2–Mub1. The Ubr2–Mub1 E3 ubiquitin ligase complex targets the transcription factor Rpn4 for proteasomal degradation. When the activity of this complex is reduced, Rpn4 accumulates, induces its own expression through PACE elements in proteasome gene promoters, and activates the *CDR1* promoter, leading to increased efflux pump expression and elevated fluconazole MICs. In *C. auris*, this mechanism has been validated by transposon mutagenesis, promoter reporter assays, and genetic complementation: the *UBR2* A316T variant identified in clinical isolates was sufficient to recapitulate the resistant phenotype through the Rpn4–CDR1 axis when introduced into a model strain (Chow et al., 2023). In our cohort, all 82 isolates carried the *UBR2* A316T substitution, and 44 of 82 isolates additionally carried an F160L variant in the same gene, whose functional significance is currently unknown. In an experimental-evolution study under fluconazole pressure, recurrent *UBR2* (P708T, H1275P) and *MUB1* (Y765*) variants emerged; subsequent allelic exchange demonstrated that these variants elevated azole MICs and increased *RPN4* and *CDR1* transcript levels, consistent with the proposed Ubr2–Mub1–Rpn4 –CDR1 model (Li et al., 2024a).

Voriconazole interpretive MIC criteria have not been established for *C. auris*. In our cohort, an in-frame deletion in *TUP1* (B9J08_03515; p.232_239delinsN, NPDFKKQT/N) was identified in 16 isolates, of which 14 (87.5%) had voriconazole MICs ≤0.5 mg/L. *TUP1* encodes a general transcriptional corepressor, but no direct experimental link between *TUP1* and azole susceptibility has been established in *C. auris*. Functional studies in this species have addressed *TUP1* mainly in the context of morphogenesis: in contrast to *C. albicans*, pseudohyphal growth in *C. auris* is independent of Tup1 (Bravo Ruiz et al., 2020), and the gene is not included among the validated azole-resistance determinants in current reviews.

Observations in related species are consistent with this pattern: in *Cryptococcus neoformans*, deletion of *TUP1* increases susceptibility to fluconazole and reduces tolerance to cell-wall stress (Lee et al., 2009). The association between the *TUP1* in-frame deletion and low voriconazole MICs in our collection should therefore be interpreted as an observational finding, pending direct experimental verification in *C. auris*.

#### 4.1.2 Mechanisms of polyene resistance in *C. auris*

Interpretation of amphotericin B susceptibility in *C. auris* is complicated by methodological factors: MIC values depend substantially on the testing protocol (EUCAST or CLSI) and platform (Vitek 2, Sensititre, Etest), and species-specific clinical breakpoints are scarce, often replaced by epidemiological cut-off values in practice (Shivarathri et al., 2022; De Luca et al., 2025). Against this backdrop, the molecular mechanisms of amphotericin B resistance in *C. auris* are insufficiently characterized—a point explicitly noted in recent transcriptomic studies and methodological comparisons. Until 2023, the only clinically validated mechanism was mutations in *ERG6*, first demonstrated *in vivo* under amphotericin B therapy, marking an initial step toward establishing a causal link between genotype and resistance phenotype in *C. auris* (Rybak et al., 2022b, 2022a). However, even in pan- or amphotericin B–resistant isolates, clear causal variants are often not identified, implying contributions from as yet unrecognized determinants and/or regulatory mechanisms, which complicates genotype–phenotype mapping in routine practice. Together with method-driven MIC discrepancies, this further exacerbates the heterogeneity of reported amphotericin B resistance rates in *C. auris* across studies. In our cohort, a single isolate (C321-E20) exceeded the EUCAST resistance breakpoint for amphotericin B (Arendrup et al., 2026); no amino-acid substitutions were detected in *ERG6* in this isolate or in any of the remaining 81, and no isolate-specific coding variants were identified in C321-E20 across the genes analyzed. This is consistent with the broader pattern that the genetic basis of polyene resistance in *C. auris* is not fully captured by mutations in known candidate genes.

Other components of the sterol pathway have also been implicated in polyene resistance. *ERG3* has been repeatedly highlighted in clinical isolates: in a Qatari clade I cohort, a premature stop-codon variant (E68*) was identified in a pan-resistant strain (CAS109) that simultaneously carried *FKS1* S639Y and novel substitutions in *CDR2*, linking *ERG3* truncation to combined amphotericin B and echinocandin resistance (Ben Abid et al., 2023). In a separate case of acquired amphotericin B resistance, a frameshift mutation in *ERG3* produced characteristic sterol remodeling, with restoration of the wild-type allele by CRISPR–Cas9 editing reducing the MIC to baseline (Carolus et al., 2024). For *ERG4*, two clade I isolates from southern Italy carried the M192I substitution with amphotericin B MICs of 2 and 4 mg/L; the authors proposed this variant as a candidate contributor to reduced polyene susceptibility, while noting the lack of functional validation (Stolfa et al., 2024). All 82 isolates in our cohort also carried *ERG4* M192I, but this substitution was present uniformly across the full range of amphotericin B MICs, including the wild-type majority and the single resistant isolate. We therefore interpret *ERG4* M192I as a clade I sublineage polymorphism rather than as a determinant of the polyene phenotype.

Transcriptional regulators acting upstream of the sterol pathway have also been linked to amphotericin B susceptibility. In a Colombian clade IV cohort, a nonsynonymous *FLO8* variant was identified and statistically associated with elevated amphotericin B MICs (Misas et al., 2019). In an *in vitro* experimental-evolution study, the amphotericin B MIC_50_ rose from 0.5 to 4 mg/L in parallel with mutations in *ERG3* and *ERG11*; allelic replacement showed that *FLO8* Q384* alone did not increase the MIC, whereas *MEC3* A272V produced an approximately two-fold elevation (Carolus et al., 2021). *FLO8* therefore acts as a context-dependent modifier rather than an independent driver of polyene resistance. In our cohort, isolate C6-E20 carried a *FLO8* frameshift variant (S408fs) and exhibited a wild-type amphotericin B MIC of 0.5 mg/L, in line with the absence of an independent effect of *FLO8* disruption in the experimental-evolution data. The same evolution study also delineated an alternative regulatory trajectory involving *UPC2*, *RTG3*, *AOX2*, and *GNP2*, with transcriptomic and metabolic remodeling accompanying MIC increases, and reported a *PRP8* T1561M substitution in this context (Carolus et al., 2021). Two isolates in our cohort (C294-E20 and C319-E20) carried *PRP8* K1662N with amphotericin B MICs of 1 mg/L. This is a different residue from T1561M and its functional consequences have not been established, so we classify it as a variant of uncertain significance. Overall, the absence of a single causative locus for amphotericin B resistance in our cohort is consistent with the emerging view that polyene resistance in *C. auris* is multifactorial and not yet fully resolved at the genetic level.

#### 4.1.3 Mechanisms of echinocandin resistance in *C. auris*

Echinocandin resistance in *C. auris* is primarily driven by amino-acid substitutions in the catalytic subunit of 1,3-β-D-glucan synthase encoded by *FKS1*. The classical resistance-conferring positions cluster in two hotspot regions of the protein: hotspot-1 (residues 635–643), where substitutions S639F, S639Y, S639P, and—less frequently—D642Y have been reported, and hotspot-2 (residues 1350–1357), with variants R1354H, R1354S, and R1354Y (Asadzadeh et al., 2022). A non-hotspot FKS1 substitution, W691L, was subsequently described in clinical isolates. CRISPR–Cas9 introduction of this allele into a susceptible background produced large MIC increases for caspofungin and micafungin (>64-fold) and a 16- to 32-fold increase for anidulafungin, confirming its causal role (Kordalewska et al., 2023). Mutations in *FKS2* are much less commonly reported in *C. auris*, although an Egyptian study identified *FKS2* F635Y in all four echinocandin-resistant isolates examined, suggesting that this paralogue can also contribute to resistance in some populations (Ahmed et al., 2025). In our cohort, all 82 isolates remained susceptible to anidulafungin and micafungin (EUCAST v.12.1), and no nonsynonymous variants were detected in either *FKS1* or *FKS2*, including their hotspot regions. This combined genotypic–phenotypic absence of echinocandin resistance is in line with global surveillance data, in which echinocandin resistance in clade I remains uncommon. Beyond *FKS1*, an experimental-evolution study of multidrug resistance in *C. auris* showed that secondary substitutions in *CIS2* (together with *ERG3*) modulated echinocandin MICs in FKS1-mutant backgrounds, suggesting an accessory role for *CIS2* (Carolus et al., 2021). The *CIS2* K74E substitution identified in all 82 of our isolates was also described in clade I isolates from Qatar (Ben Abid et al., 2023) and in the Austrian collection (Spettel et al., 2023), in both cases without an associated echinocandin-resistant phenotype, supporting the interpretation that *CIS2* K74E represents a clade I polymorphism rather than an independent resistance determinant.

Cell-wall stress signaling can also modulate baseline echinocandin tolerance independently of the target enzyme. In *C. auris*, deletion of *SSK1*—which encodes a two-component response regulator upstream of the *Hog1* MAPK pathway—restores susceptibility to caspofungin and amphotericin B, indicating that an intact Ssk1–Hog1 axis supports tolerance to both drug classes (Shivarathri et al., 2020). Two isolates in our cohort, C285-E20 and C320-E20, carried an *SSK1* K583Q substitution. To our knowledge, this variant has not been previously reported in *C. auris*. Both carrier isolates exhibited wild-type echinocandin MICs, suggesting that the variant does not by itself confer resistance in this genetic background. Its broader functional significance, including possible effects on tolerance or persistence under sub-MIC drug exposure, would need to be tested directly by allelic replacement followed by MIC determination.

#### 4.1.4 Mechanisms of 5-flucytosine resistance in *C. auris*

In our cohort, premature stop codons in *FCY2* were the most common molecular event among 5-FC–resistant isolates. The L383* variant (T1148G) was detected in 17 isolates—three by whole-genome sequencing (C285-E20, C310-E20, and C320-E20) and 14 by targeted Sanger sequencing—and an additional truncating variant, S328*, was identified in one isolate (C697-E20). Sixteen of the seventeen L383*-carrying isolates and the single S328*-carrying isolate displayed 5-FC MICs of 128 mg/L. The remaining L383* carrier, C320-E20, exhibited a substantially lower 5-FC MIC of 0.25 mg/L—slightly above the 0.06–0.125 mg/L baseline observed among susceptible isolates but far below the 128 mg/L measured for the other FCY2-truncated isolates—indicating incomplete genotype–phenotype concordance for this variant. The low 5-FC MIC of C320-E20 may reflect partial compensation for impaired Fcy2 function by another FCY2-like transporter, allowing sufficient drug uptake to maintain susceptibility. Differences in the expression or activity of downstream components of the 5-FC activation pathway may also modulate the phenotypic effect of L383*. Functional studies will be required to distinguish between these possibilities. Thus, although *FCY2* truncation was strongly associated with high-level 5-FC resistance in most isolates, L383* alone may not be sufficient as a standalone molecular predictor of resistance.

The next step in 5-FC activation is the intracellular deamination of 5-FC to 5-fluorouracil (5-FU) by cytosine deaminase Fcy1. Loss-of-function variants of *FCY1* have been established as a route to 5-FC resistance in other *Candida* species, including *C. dubliniensis* (McManus et al., 2009), but in *C. auris* no specific *FCY1* allele has yet been functionally validated as causal for high-level resistance. The *FCY1* S70R substitution previously identified in serial New York clinical isolates illustrates the limits of inference based on prevalence alone: it was present in all isolates from those patients before any exposure to 5-FC and was not associated with elevated MICs (Jacobs et al., 2022). In our cohort, the same S70R variant was carried by all 82 isolates, spanning the full range of 5-FC MICs, supporting its interpretation as a neutral clade I polymorphism rather than a contributor to resistance.

Conversion of 5-FU to its active nucleotide derivatives requires the uracil phosphoribosyltransferase Fur1, which generates 5-fluorouridine monophosphate; downstream products disrupt RNA and DNA synthesis and arrest fungal growth. Disruption of *FUR1* is a well-established route to 5-FC resistance in yeasts (Papon et al., 2007; Frías-De-León et al., 2020). In *C. auris*, experimental selection under 5-FC rapidly generates nonsense (Q30*) and frameshift *FUR1* variants with 5-FC MICs ≥64 mg/L and near-complete loss of susceptibility (Phan-Canh et al., 2025), and a 33-residue in-frame deletion in FUR1 has been described in a pan-resistant clinical New York isolate with both 5-FC resistance and 5-FU cross-resistance (Jacobs et al., 2022). A missense *FUR1* F211I variant was reported during a nosocomial outbreak investigation, although its functional consequence has not been established (Rhodes et al., 2018). In our cohort, isolate C245-E20 carried a 28-residue in-frame deletion in *FUR1* (p.Phe167_Ile194del) in the absence of *FCY2* mutations and displayed a 5-FC MIC of 128 mg/L, consistent with loss of uracil phosphoribosyltransferase function. Treatment records for the patients from whom 5-FC–resistant isolates were recovered were not available, so the contribution of treatment-driven selection to the observed mutational landscape cannot be assessed in this dataset.

A further candidate locus is *ADE17*, which encodes AICAR transformylase in the purine biosynthesis pathway. A New York clinical series reported the combination *ADE17* G45V with FUR1 Δ33 in 5-FC–resistant *C. auris* isolates, raising the possibility that alterations in purine metabolism modulate fluoropyrimidine toxicity (Jacobs et al., 2022); a direct functional role for *ADE17* in 5-FC resistance has not yet been demonstrated. No coding variants in *ADE17* were detected in our cohort, including in the four isolates with elevated 5-FC MICs.

Beyond the canonical *FCY2*–*FCY1*–*FUR1* pathway that governs 5-FC transport and activation, the response of *C. auris* to this drug appears to be influenced by tolerance to genotoxic stress mediated by cell-cycle checkpoints. An experimental study showed that deletion of *MRC1* (Mediator of Replication Checkpoint 1) in *C. auris* increases susceptibility to inducers of replication stress—hydroxyurea (HU) and methyl methanesulfonate (MMS)—as well as to 5-FC; it is accompanied by S-phase prolongation and defects in checkpoint activation, indicating a supportive role of an intact S-phase response in survival under 5-FC exposure (Bravo Ruiz et al., 2020). In our dataset, *MRC1* Q313R was identified in 81 of 82 isolates (98.8%). Given its near-uniform distribution across the cohort and absence of an established functional link to 5-FC susceptibility, we interpret this variant as a clade-associated polymorphism rather than a resistance determinant; isogenic allele-editing in a clade I background would be required to test this directly. Nevertheless, its consistent presence underscores the need for further functional testing of *MRC1* variants in relevant clinical genetic contexts.

## 5 Genomic variants in *C. auris* potentially associated with pan-resistance

Beyond the mutational patterns already discussed that are associated with resistance to the main classes of antifungal agents, there is a set of specific genomic variants linked to the development of pan-resistance. These genetic alterations are of particular interest to contemporary mycology, as they determine the ability of yeast to withstand the effects of multiple antifungal classes simultaneously. Analysis of clinical data identified recurrent substitutions *URK1* (S472V), *YEN1* (A294V), and *URB2* (L641F) in pan-resistant *C. auris* isolates recovered during the natural course of infection (Jacobs et al., 2022). The frequency of these variants in the global *C. auris* population remains undetermined; to date, they have been reported primarily in isolates from the South Asian clade (Clade I). In our collection (n = 82), all isolates carry these substitutions, suggesting that they may represent a clade-specific polymorphism rather than a universal marker of pan-resistance.

## 6 Global phylogenetic relationships and local transmission patterns of *C. auri*s in Russia: an evolutionary and epidemiological perspective

Our phylogenomic analysis places the Russian *C. auris* population within clade I and shows that, despite inclusion of a broad international comparison set, the Russian isolates occupy a relatively restricted segment of global diversity rather than being scattered across multiple distant sublineages. In this context, the adjacency of the Russian cluster to isolates from Italy and the USA is best interpreted as evidence of its placement within the globally disseminated clade I background, rather than as direct proof of a specific importation route. This distinction is important because recent population-genomic studies have shown increasing phylogeographic mixing within *C. auris*, particularly in clade I, with national outbreaks often reflecting a combination of international introduction, local transmission (Chow et al., 2020; Di Pilato et al., 2021).

The phylogenomic structure of the Russian cohort indicates that *C. auris* was not confined to isolated hospital outbreaks. Isolates from different institutions were interspersed within closely related sublineages, indicating broader circulation of related genotypes across the healthcare system. Specific transmission chains cannot be reconstructed without dedicated epidemiological metadata, but this pattern is consistent with the established capacity of *C. auris* to persist in healthcare environments and to spread through patient-care networks.

A comparable structure has been described in Qatar, where 76 C*. auris* isolates from nine major hospitals collected during the COVID-19 period formed a highly clonal clade I population (Ben Abid et al., 2023). The Qatari population diverges from ours in the principal *ERG11* mutation (Y132F vs our K143R) but shares the same set of secondary substitutions in *ERG4*, *CIS2*, *SNQ2*, and *CDR1*, suggesting that the two populations occupy adjacent positions within clade I and are subject to a similar epidemiological pattern: establishment of *C. auris* in healthcare environments, persistence over prolonged periods, and dissemination through networks of institutions linked by patient transfers and shared clinical pathways.

In the Russian dataset, this pattern is reflected in the geographic structuring of the population around two major urban healthcare networks, in Saint Petersburg and Moscow. These lineages represent regional rather than institutional circulation, with hospitals in the two cities acting as central nodes connected to other facilities through patient-care pathways. Quantitatively, however, the Moscow population was more internally diverse than the Saint Petersburg population; the larger Saint Petersburg cluster therefore appears more branched on the tree mainly because it contains more isolates, whereas its strains are on average more closely related to one another. Overall, the phylogenomic structure is consistent with local establishment of clade I *C. auris* after introduction and subsequent circulation within healthcare networks, rather than with a series of independent importation events.

Time-calibrated phylogenetic analysis reframes the question of *C. auris* emergence: rather than identifying when the organism was first detected, it asks how long a lineage had already been circulating before it became clinically visible. This question is particularly relevant for *C. auris*, whose epidemiology is shaped by prolonged skin colonization, environmental persistence, incomplete screening, and delayed laboratory recognition, all of which can decouple clinical recognition from actual emergence. Global molecular-clock analyses have shown that outbreak-associated clusters within clades I, III, and IV diverged decades before their broad clinical detection (Chow et al., 2020). In the Russian cohort, the most recent common ancestor of the 82 sampled isolates was placed at approximately 2008, and the major internal node giving rise to most currently sampled lineages at around 2014, both predating the first laboratory-confirmed Russian case. The dated phylogeny therefore indicates that the lineage represented in our collection had already diversified into multiple descendant lineages before its clinical recognition, consistent with the broader observation that the appearance of *C. auris* in a national surveillance system marks the point at which an existing transmission system becomes visible rather than the start of local circulation.

The phylogenetic patterns observed in our Russian cohort, together with those reported from other countries, indicate that *C. auris* surveillance is not well captured by a hospital-by-hospital framework. Once *C. auris* becomes established in a region, it can circulate across a connected healthcare environment rather than remain confined to a single institution: patient transfers, prolonged admissions, and shared clinical pathways link individual hospitals, regional centers, and long-term care facilities into a single epidemiological network. Bloodstream isolates, on which most clinical attention is focused, represent only the most visible part of this circulating population; a substantially larger reservoir is plausible among colonized patients and within the hospital environment itself. Effective control therefore depends on integrating genomic surveillance with active colonization screening, environmental sampling, and structured information on patient movement between institutions, since only this combination can distinguish direct transmission from exposure to a shared reservoir or repeated acquisition within the same healthcare network.

## 7 Limitations

This study has several limitations. The analyzed dataset included 82 isolates sequenced within a single Illumina NextSeq 550 run, which provided consistency in sequencing conditions but limited the sample size. Therefore, the findings should be interpreted with caution when extrapolating them to the broader population structure of *C. auris* in Russia and neighboring regions. In addition, the study was based on short-read whole-genome sequencing, which may have limited the detection of large structural variants, copy number variations, and complex genomic rearrangements. The role of identified mutations in antifungal resistance was inferred from genomic data and previously published evidence, without functional validation. Finally, limited clinical metadata for some isolates restricted the analysis of associations between genomic features, treatment history, infection control measures, and patient outcomes.

## Data Availability

The raw whole-genome sequencing reads generated in this study have been deposited in the NCBI Sequence Read Archive (SRA) under BioProject accession number PRJNA1504518. The BioProject has been released and contains 82 linked BioSample records and 82 linked SRA records. The targeted FCY2 gene sequences have been deposited in NCBI GenBank under accession numbers PZ822450 -PZ822467.

## Supplementary material

Supplementary Figure 1 Global phylogenetic context of Russian *C. auris* isolates. Maximum-likelihood phylogenetic tree of 423 C*. auris* genomes representing all recognized clades, rooted using [SRR9007776] outgroup sequence (SRA accession SRR9007776). The tree was inferred in IQ-TREE under the best-fit substitution model selected by ModelFinder. Branch support values ​​are shown at internal nodes as SH-aLRT (%)/UFBoot (%). The outer ring indicates clade assignment (clades I–VI; see legend); the inner ring indicates country of origin. Russian isolates (C-prefixed labels) form a single cluster within clade I (the South Asian clade).

Supplementary Figure 2 Pairwise genomic distances between Russian isolates and international reference populations. Boxplots show pairwise SNP distances between *C. auris* isolates from Moscow (n = 24) and St. Petersburg (n = 58) and isolates from seven selected countries: the five countries with the lowest median pairwise SNP distance from Moscow isolates (Italy, USA, Mexico, Kuwait, Saudi Arabia), plus two additional geographically distant reference countries (India, South Africa), as described in Methods. Each point represents a single pairwise comparison; boxes indicate the interquartile range (IQR), the central line shows the median, and whiskers extend to 1.5× IQR.

Supplementary Table 1 Characteristics of the 15 additional *C. auris* isolates included in the targeted *FCY2* sequencing cohort, including collection date, hospital, specimen type, 5-flucytosine MIC, and the corresponding *FCY2* nucleotide and predicted amino-acid change.

Supplementary Table 2 Metadata for 341 publicly available *C. auris* genomes downloaded from the NCBI Sequence Read Archive (SRA) and used for comparative phylogenomic analysis, including clade assignment, country of origin, BioProject/BioSample accessions, and sequencing platform.

Supplementary Table 3 Oligonucleotide primers used for PCR amplification and Sanger sequencing of the *FCY2* gene in *C. auris*.

Supplementary Table 4 Minimum inhibitory concentrations (MIC, mg/L) of nine antifungal agents for all 82 clinical *C. auris* isolates included in whole-genome sequencing.

Supplementary Table 5 List of 74 candidate genes previously implicated in antifungal resistance in *Candidozyma auris*, compiled through literature review, with corresponding genomic coordinates in reference genome assemblies GCA_002759435.2 (V2) and GCA_002759435.3 (V3).

Supplementary Table 6 Complete list of variants identified within the 74 candidate resistance genes across the 82 Russian *C. auris* isolates, including synonymous and noncoding substitutions.
