# Supplementary material for: Resistance gene mutations and phylogenetic relationships in Candidozyma auris isolates from Russia
Source: Front Cell Infect Microbiol. 2026 Aug 28;16:1929119. doi: 10.3389/fcimb.2026.1929119 (PMC13561818; doi:10.3389/fcimb.2026.1929119)
Supplement: Supplementary file 1 [file Table1.docx]

**Supplementary Table S1. Characteristics of the 15 *Candidozyma auris* isolates included in the targeted *FCY2* sequencing cohort.**

| **Isolate ID** | **Collection date** | **Hospital** | **Specimen** | **5-FC MIC (mg/L)** | ***FCY2* nucleotide variant** | **Predicted amino acid change** |
| --- | --- | --- | --- | --- | --- | --- |
| **C374-E20** | 09.06.2022 | 10 | Urine | 128 | T1148G | L383* |
| **C682-E20** | 05.03.2024 | 17 | Blood | 128 | T1148G | L383* |
| **C683-E20** | 11.03.2024 | 17 | Pleural fluid | 128 | T1148G | L383* |
| **C684-E20** | 22.05.2024 | 17 | Blood | 128 | T1148G | L383* |
| **C686-E20** | 09.05.2024 | 17 | Blood | 128 | T1148G | L383* |
| **C687-E20** | 20.05.2024 | 17 | BAL | 128 | T1148G | L383* |
| **C688-E20** | 05.03.2024 | 17 | BAL | 128 | T1148G | L383* |
| **C689-E20** | 19.05.2024 | 17 | Wound | 128 | T1148G | L383* |
| **C690-E20** | 04.04.2024 | 17 | BAL | 128 | T1148G | L383* |
| **C691-E20** | 20.05.2024 | 17 | BAL | 128 | T1148G | L383* |
| **C692-E20** | 02.04.2024 | 17 | Wound | 128 | T1148G | L383* |
| **C694-E20** | 21.04.2024 | 17 | BAL | 128 | T1148G | L383* |
| **C695-E20** | 11.04.2024 | 17 | Wound | 128 | T1148G | L383* |
| **C696-E20** | 09.04.2024 | 17 | BAL | 128 | T1148G | L383* |
| **C697-E20** | 17.06.2024 | 19 | Urine | 128 | C983A | S328* |
