# Supplementary material for: Resistance gene mutations and phylogenetic relationships in Candidozyma auris isolates from Russia
Source: Front Cell Infect Microbiol. 2026 Aug 28;16:1929119. doi: 10.3389/fcimb.2026.1929119 (PMC13561818; doi:10.3389/fcimb.2026.1929119)
Supplement: Supplementary file 2 [file Table2.docx]

| **Name** | **Sequence** | **Length (bp)** |
| --- | --- | --- |
| **For1** | GCATTCTGCAAGTACGCGAG | 20 |
| **For2** | TCCATATTTGGGTGCCGCTT | 20 |
| **For3** | CGTGGGAGGCCTTGTTTACT | 20 |
| **For4** | TCTGCTGTGGTGTTGCTGGT | 20 |
| **Rev1** | ACTATAGTAGGCACTATCTAC | 21 |
| **Rev2** | TACCACACCTGGTTCATGCC | 20 |
| **Rev3** | CACAAGAACTGGCCAAAGCC | 20 |
| **Rev4** | CCAAAATCAAGCACCCAGCC | 20 |
