# Supplementary material for: Resistance gene mutations and phylogenetic relationships in Candidozyma auris isolates from Russia
Source: Front Cell Infect Microbiol. 2026 Aug 28;16:1929119. doi: 10.3389/fcimb.2026.1929119 (PMC13561818; doi:10.3389/fcimb.2026.1929119)
Supplement: Supplementary file 3 [file Table3.docx]

**Supplementary Table S4 Minimum inhibitory concentrations (MIC, mg/mL) of nine antifungal agents for clinical isolates of *Candidozyma auris*.**

| Strain ID | AmB | VOR | ITR | PZ | FCZ | AND | CAS | MCF | 5-FC |
| --- | --- | --- | --- | --- | --- | --- | --- | --- | --- |
| С1-E20 | 0.5 | 2 | 0.25 | 0.12 | 64 | 0.06 | 0.12 | 0.03 | 0.06 |
| С2-E20 | 0.5 | 4 | 0.12 | 0.12 | 64 | 0.06 | 0.06 | 0.06 | 0.06 |
| С3-E20 | 0.25 | 4 | 0.25 | 0.12 | 64 | 0.015 | 0.25 | 0.12 | 0.06 |
| С4-E20 | 0.5 | 2 | 0.12 | 0.12 | 64 | 0.06 | 0.06 | 0.03 | 0.06 |
| С6-E20 | 0.5 | 2 | 0.12 | 0.12 | 64 | 0.12 | 0.12 | 0.06 | 0.06 |
| С8-E20 | 0.5 | 1 | 0.12 | 0.12 | 64 | 0.06 | 0.12 | 0.06 | 0.06 |
| С29-E20 | 0.5 | 0.5 | 0.12 | 0.12 | 64 | 0.06 | 0.12 | 0.06 | 0.06 |
| С30-E20 | 0.5 | 2 | 0.12 | 0.12 | 64 | 0.06 | 0.12 | 0.03 | 0.06 |
| С40-E20 | 1 | 2 | 0.12 | 0.12 | 64 | 0.12 | 0.06 | 0.12 | 0.06 |
| С41-E20 | 0.5 | 2 | 0.5 | 0.12 | 64 | 0.12 | 0.12 | 0.12 | 0.06 |
| С42-E20 | 1 | 2 | 0.12 | 0.12 | 64 | 0.06 | 0.12 | 0.12 | 0.06 |
| С49-E20 | 0.5 | 2 | 0.12 | 0.12 | 64 | 0.06 | 0.12 | 0.12 | 0.06 |
| С56-E20 | 0.5 | 2 | 0.12 | 0.12 | 64 | 0.015 | 0.12 | 0.06 | 0.06 |
| С57-E20 | 0.5 | 2 | 0.12 | 0.12 | 64 | 0.12 | 0.12 | 0.12 | 0.06 |
| С65-E20 | 1 | 0.12 | 0.06 | 0.03 | 32 | 0.03 | 0.12 | 0.06 | 0.06 |
| С67-E20 | 0.5 | 0.12 | 0.015 | 0.03 | 16 | 0.12 | 0.12 | 0.03 | 0.06 |
| С69-E20 | 1 | 0.12 | 0.015 | 0.12 | 16 | 0.03 | 0.06 | 0.12 | 0.06 |
| С70-E20 | 0.5 | 2 | 0.12 | 0.12 | 64 | 0.12 | 0.12 | 0.06 | 0.06 |
| С92-E20 | 0.5 | 4 | 0.25 | 0.12 | 64 | 0.015 | 0.12 | 0.06 | 0.12 |
| С103-E20 | 1 | 2 | 0.12 | 0.12 | 64 | 0.03 | 0.12 | 0.06 | 0.06 |
| С107-E20 | 0.5 | 4 | 0.12 | 0.12 | 64 | 0.12 | 0.12 | 0.06 | 0.06 |
| С108-E20 | 1 | 2 | 0.12 | 0.12 | 64 | 0.015 | 0.12 | 0.03 | 0.12 |
| С113-E20 | 0.5 | 2 | 0.06 | 0.12 | 64 | 0.12 | 0.12 | 0.06 | 0.06 |
| С117-E20 | 1 | 4 | 0.12 | 0.12 | 64 | 0.12 | 0.12 | 0.06 | 0.06 |
| С121-E20 | 1 | 0.5 | 0.12 | 0.12 | 64 | 0.12 | 0.25 | 0.03 | 0.06 |
| С125-E20 | 0.5 | 2 | 0.25 | 0.12 | 64 | 0.06 | 0.12 | 0.03 | 0.06 |
| С127-E20 | 1 | 2 | 0.12 | 0.12 | 64 | 0.12 | 0.12 | 0.03 | 0.06 |
| С131-E20 | 1 | 2 | 0.12 | 0.12 | 64 | 0.015 | 0.12 | 0.03 | 0.06 |
| С155-E20 | 1 | 2 | 0.12 | 0.12 | 64 | 0.015 | 0.12 | 0.12 | 0.06 |
| С168-E20 | 1 | 2 | 0.25 | 0.12 | 64 | 0.12 | 0.12 | 0.12 | 0.06 |
| С172-E20 | 1 | 0.03 | 0.015 | 0.008 | 32 | 0.03 | 0.06 | 0.03 | 0.06 |
| С175-E20 | 1 | 4 | 0.25 | 0.12 | 64 | 0.12 | 0.06 | 0.12 | 0.06 |
| С182-E20 | 1 | 2 | 0.5 | 0.12 | 64 | 0.12 | 0.12 | 0.06 | 0.12 |
| С193-E20 | 0.5 | 2 | 0.12 | 0.12 | 64 | 0.12 | 0.12 | 0.06 | 0.12 |
| С199-E20 | 2 | 2 | 0.12 | 0.12 | 64 | 0.25 | 0.06 | 0.12 | 0.12 |
| С202-E20 | 1 | 4 | 0.25 | 0.12 | 64 | 0.06 | 0.12 | 0.12 | 0.12 |
| С210-E20 | 1 | 0.5 | 0.25 | 0.12 | 64 | 0.06 | 0.12 | 0.12 | 0.06 |
| С222-E20 | 0.5 | 2 | 0.25 | 0.12 | 64 | 0.06 | 0.12 | 0.12 | 0.06 |
| С225-E20 | 1 | 2 | 0.12 | 0.12 | 64 | 0.03 | 0.06 | 0.03 | 0.06 |
| С227-E20 | 0.5 | 2 | 0.5 | 0.12 | 64 | 0.03 | 0.03 | 0.06 | 0.06 |
| С230-E20 | 0.5 | 0.03 | 0.015 | 0.12 | 16 | 0.06 | 0.12 | 0.12 | 0.12 |
| С234-E20 | 1 | 2 | 0.25 | 0.12 | 64 | 0.06 | 0.12 | 0.12 | 0.12 |
| С237-E20 | 0.5 | 0.03 | 0.06 | 0.015 | 32 | 0.06 | 0.12 | 0.12 | 0.06 |
| С240-E20 | 2 | 0.03 | 0.12 | 0.03 | 16 | 0.12 | 0.06 | 0.06 | 0.06 |
| С241-E20 | 1 | 0.015 | 0.015 | 0.015 | 16 | 0.015 | 0.06 | 0.12 | 0.06 |
| С245-E20 | 0.5 | 0.5 | 0.12 | 0.12 | 64 | 0.12 | 0.12 | 0.03 | 128 |
| С248-E20 | 0.5 | 0.5 | 0.12 | 0.12 | 64 | 0.12 | 0.12 | 0.06 | 0.12 |
| С257-E20 | 1 | 0.06 | 0.06 | 0.03 | 16 | 0.03 | 0.06 | 0.12 | 0.06 |
| С262-E20 | 0.5 | 2 | 0.25 | 0.12 | 64 | 0.12 | 0.25 | 0.12 | 0.12 |
| С264-E20 | 1 | 2 | 0.25 | 0.12 | 64 | 0.12 | 0.12 | 0.12 | 0.12 |
| С265-E20 | 1 | 2 | 0.25 | 0.12 | 64 | 0.12 | 0.12 | 0.06 | 0.12 |
| С273-E20 | 1 | 2 | 0.12 | 0.12 | 64 | 0.06 | 0.12 | 0.06 | 0.06 |
| С274-Е20 | 0.5 | 1 | 0.12 | 0.12 | 64 | 0.06 | 0.12 | 0.06 | 0.06 |
| С275-E20 | 1 | 0.5 | 0.25 | 0.12 | 64 | 0.12 | 0.06 | 0.06 | 0.06 |
| С277-E20 | 1 | 2 | 0.25 | 0.12 | 64 | 0.06 | 0.12 | 0.06 | 0.12 |
| С279-E20 | 1 | 2 | 0.5 | 0.12 | 64 | 0.06 | 0.12 | 0.12 | 0.06 |
| С284-E20 | 1 | 2 | 0.06 | 0.12 | 64 | 0.06 | 0.12 | 0.12 | 0.12 |
| С285-E20 | 1 | 1 | 0.015 | 0.03 | 64 | 0.015 | 0.12 | 0.12 | 128 |
| С287-E20 | 1 | 2 | 0.25 | 0.12 | 64 | 0.06 | 0.06 | 0.06 | 0.06 |
| С289-E20 | 1 | 1 | 0.25 | 0.12 | 64 | 0.015 | 0.12 | 0.12 | 0.06 |
| С294-E20 | 1 | 2 | 0.06 | 0.12 | 64 | 0.12 | 0.12 | 0.12 | 0.06 |
| С300-E20 | 1 | 2 | 0.06 | 0.12 | 64 | 0.06 | 0.12 | 0.06 | 0.06 |
| С302-E20 | 1 | 1 | 0.12 | 0.12 | 64 | 0.03 | 0.06 | 0.12 | 0.12 |
| С305-E20 | 1 | 1 | 0.015 | 0.12 | 64 | 0.06 | 0.06 | 0.03 | 0.12 |
| С310-E20 | 1 | 1 | 0.25 | 0.12 | 64 | 0.12 | 0.06 | 0.12 | 128 |
| С315-E20 | 2 | 2 | 0.12 | 0.03 | 64 | 0.25 | 0.12 | 0.12 | 0.06 |
| С317-E20 | 1 | 1 | 0.12 | 0.12 | 64 | 0.06 | 0.12 | 0.06 | 0.06 |
| С318-E20 | 1 | 1 | 0.12 | 0.12 | 64 | 0.06 | 0.12 | 0.06 | 0.06 |
| С319-E20 | 1 | 2 | 0.25 | 0.12 | 64 | 0.06 | 0.12 | 0.06 | 0.06 |
| С320-E20 | 2 | 4 | 0.25 | 0.12 | 64 | 0.06 | 0.12 | 0.06 | 0.25 |
| С321-E20 | 4 | 2 | 0.25 | 0.12 | 64 | 0.12 | 0.12 | 0.06 | 0.06 |
| С349-E20 | 1 | 2 | 0.12 | 0.03 | 64 | 0.06 | 0.06 | 0.03 | 0.06 |
| С351-E20 | 0.5 | 1 | 0.12 | 0.03 | 64 | 0.12 | 0.12 | 0.12 | 0.06 |
| С356-E20 | 1 | 2 | 0.12 | 0.12 | 64 | 0.03 | 0.12 | 0.12 | 0.06 |
| С359-E20 | 1 | 2 | 0.12 | 0.12 | 64 | 0.03 | 0.12 | 0.12 | 0.06 |
| С390-E20 | 2 | 2 | 0.12 | 0.12 | 64 | 0.06 | 0.12 | 0.06 | 0.06 |
| С399-E20 | 1 | 0.015 | 0.015 | 0.008 | 8 | 0.06 | 0.06 | 0.06 | 0.06 |
| С410-E20 | 2 | 0.12 | 0.12 | 0.008 | 8 | 0.06 | 0.12 | 0.06 | 0.06 |
| С457-E20 | 2 | 2 | 0.12 | 0.03 | 64 | 0.12 | 0.06 | 0.12 | 0.06 |
| С512-E20 | 1 | 1 | 0.12 | 0.12 | 64 | 0.015 | 0.06 | 0.03 | 0.06 |
| С536-E20 | 2 | 1 | 0.25 | 0.12 | 64 | 0.25 | 0.12 | 0.06 | 0.06 |
| С537-E20 | 2 | 0.5 | 0.25 | 0.12 | 64 | 0.12 | 0.06 | 0.12 | 0.06 |
